# Forest Structure, Stand Composition, and Climate-Growth Response in Montane Forests of Jiuzhaigou National Nature Reserve, China

**DOI:** 10.1371/journal.pone.0071559

**Published:** 2013-08-09

**Authors:** Mark W. Schwartz, Christopher R. Dolanc, Hui Gao, Sharon Y. Strauss, Ari C. Schwartz, John N. Williams, Ya Tang

**Affiliations:** 1 Department of Environmental Science and Policy, University of California Davis, Davis, California, United States of America; 2 Department of Forest Management, University of Montana, Missoula, Montana, United States of America; 3 Department of Environment, Sichuan University Chengdu, Sichuan, Peoples Republic of China; 4 Department of Evolution and Ecology, University of California Davis, Davis, California, United States of America; 5 Department of Biology, Oberlin College, Oberlin, Ohio, United States of America; DOE Pacific Northwest National Laboratory, United States of America

## Abstract

Montane forests of western China provide an opportunity to establish baseline studies for climate change. The region is being impacted by climate change, air pollution, and significant human impacts from tourism. We analyzed forest stand structure and climate-growth relationships from Jiuzhaigou National Nature Reserve in northwestern Sichuan province, along the eastern edge of the Tibetan plateau. We conducted a survey to characterize forest stand diversity and structure in plots occurring between 2050 and 3350 m in elevation. We also evaluated seedling and sapling recruitment and tree-ring data from four conifer species to assess: 1) whether the forest appears in transition toward increased hardwood composition; 2) if conifers appear stressed by recent climate change relative to hardwoods; and 3) how growth of four dominant species responds to recent climate. Our study is complicated by clear evidence of 20^th^ century timber extraction. Focusing on regions lacking evidence of logging, we found a diverse suite of conifers (*Pinus, Abies, Juniperus, Picea*, and *Larix*) strongly dominate the forest overstory. We found population size structures for most conifer tree species to be consistent with self-replacement and not providing evidence of shifting composition toward hardwoods. Climate-growth analyses indicate increased growth with cool temperatures in summer and fall. Warmer temperatures during the growing season could negatively impact conifer growth, indicating possible seasonal climate water deficit as a constraint on growth. In contrast, however, we found little relationship to seasonal precipitation. Projected warming does not yet have a discernible signal on trends in tree growth rates, but slower growth with warmer growing season climates suggests reduced potential future forest growth.

## Introduction

Global change impacts on climate and air quality are driving potentially major ecosystem responses even in remote ecosystems [1]. Failure to detect signals of ecosystem drivers could lead to a process of system change with negative consequences for long range protection of resources in remote sites. Any impact that climate change has on China’s more than 207 million ha of forest (5^th^ largest forest area globally [2]) could have global consequences [3]–[9].

To better understand the potential impacts of climate change on forest composition and tree growth in Chinese forests, we combined research on forest structure with a dendroecological analysis of growth response to temperature and rainfall for the montane forests of Jiuzhaigou National Nature Reserve (JNNR). Although there have been relatively few vegetation studies conducted in JNNR, it offers a range of conditions amenable to conducting such research, including varied topography, variation in human density and impact, and diverse plant community types. In particular, the numerous vegetation zones found in close proximity to one another across steep elevation and primary productivity gradients make JNNR an excellent backdrop against which to test for evidence of climate change driving vegetation responses. Evidence from two centuries of tree-ring data suggests that forests in the region are responsive to climate variability [10]–[12], and that climate change, as indicated by a warming trend through the latter half of the 20^th^ century, may already be occurring [13].

In this study, we used a replicable, cost-effective rapid assessment approach to address three research objectives. First, we evaluated whether forests in JNNR appear to show an increasing prevalence of hardwood species. Mid-twentieth century logging in the region resulted in the rapid regeneration of conifers. As these forests mature, however, there is speculation that the balance between hardwoods and conifers is shifting. Some models predict that with warming trends and a lack of disturbance, hardwoods will displace conifers in many temperate forests under climate change [14], [15]. Evidence of this transition has been observed in the Green Mountains in Vermont, USA [16], and a recent model of climate change-driven vegetation dynamics predicts China will lose upwards of two thirds of its cool conifer forest, taiga and tundra vegetation, while cool mixed forest was predicted to increase in area [17].

In this study, we quantified tree composition in a series of forest plots to determine if a similar phenomenon is occurring for the common conifer species of the JNNR. Second, we tested for evidence of a climatic gradient in forest change. That is, if a transition in forest structure and composition is occurring, we wanted to determine whether the extent of change was dependent on the position of the forest along a 1300 m elevation gradient. Third, we assessed the potential for forests to change in the future in response to changing climate by correlating annual tree-ring growth for a suite of conifer species with recent climatic data for the study region. We then compared these results to similar studies in other montane regions of China. This assessment allows us to predict how forests may respond to future climate change and which taxa might be favored by such change.

## Study Area

**Figure 1:**
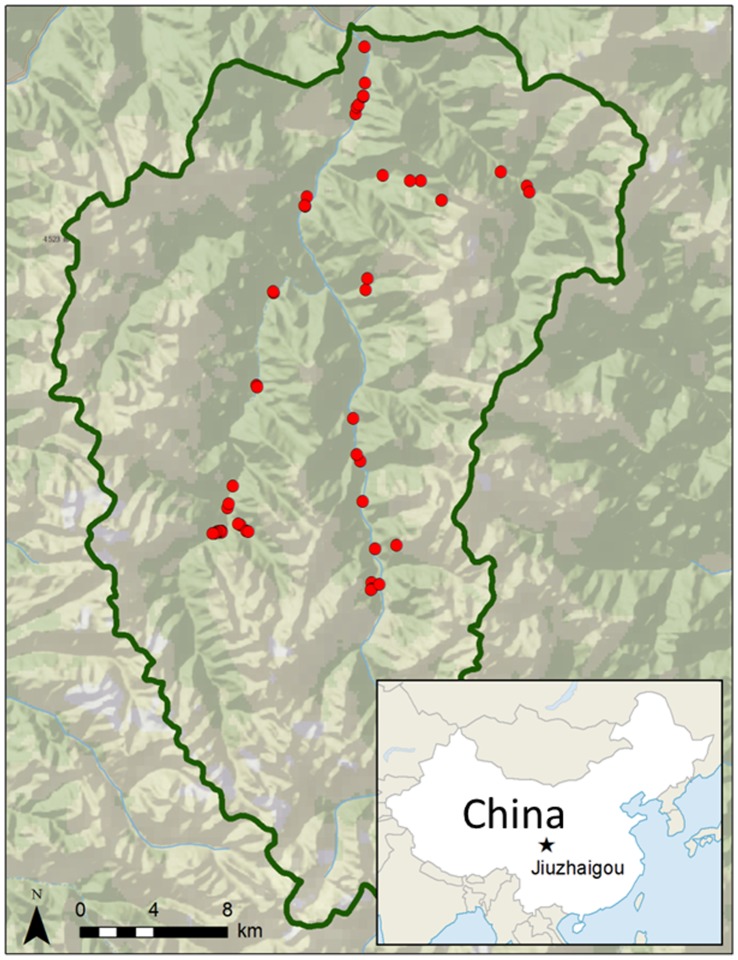
A map of Jiuzhaigou National Nature Reserve. The map shows plot sampling locations (red dots), and location of Reserve in the context of China (inset).

Situated in China’s northern Sichuan province close to the southern border of Gansu Province, JNNR (Fig. 1) lies in a transitional region from the humid Sichuan basin to the semiarid Tibetan plateau at the edge of a monsoonal-influenced climatic zone [18]–[21]. The Reserve consists of high mountain valleys surrounded by the peaks of the Min Shan mountain range. With an elevation range spanning from 2000 to 4800 m, JNNR includes several major vegetation zones, from cool mixed forest at low elevations through cool conifer forest and taiga at mid elevations, to tundra at the upper elevations [22]. Designated as an IUCN World Biosphere Reserve in 1997, JNNR is also characterized by a rich diversity of both deciduous and coniferous forest types with sixteen taxa in the Pinaceae [21]. Intermixed with the conifers are hardwoods from genera typical of mixed conifer forests (e.g., *Betula, Populus, Salix*), but also with hardwood genera more common to temperate forests (e.g., *Quercus*, *Acer, Sorbus*) [21].

The Reserve contains nine traditional villages, of which most are currently occupied. As a consequence of park-imposed constraints on agricultural practices within the park, traditional impacts on forests through coppicing hardwoods for fuel appear to be decreasing. This does not, however, mean that human impacts are decreasing. JNNR is transitional from the densely populated Sichuan basin to the sparsely populated Tibetan plateau, creating a steep gradient with respect to anthropogenic drivers of environmental impact, including acid rain impacts [19] and N deposition [18]. With over two million visitors each year, tourism also results in significant impacts to parts of the Reserve, such as the Jiuzhaigou Valley where people go to see the lakes and geological formations [23]–[27].

## Methods

Our work was conducted within JNNR at the behest of the Reserve Authority. All travel within JNNR was done in reserve vehicles, driven by employees, with full knowledge and consent of our project. The study included no animal collection and did not deal with endangered species. Plant tissue samples for field identification were left with the park herbarium.

### Vegetation Plot Sampling

Our goal was to use a rapid sampling protocol to establish baseline data describing forest structure and composition within the main forested regions of JNNR. We sampled plots (Fig. 1) with approximately equal effort across each of JNNR’s three main river valleys (Rize, Zechawa and Shuzeng) accessible by road or trail. An effort was made to capture the main differences in topography and to distribute the plots roughly equally across the elevation range selected. Plots were generally located close to roads and trails. Generalized plot locations were selected in areas of uniform vegetation (i.e., not crossing any sharp ecotones). Plot centers were determined by a random flag toss within these generally uniform sampling areas.

We sampled forest structure in JNNR using circular plots of 15 m radius (707 m^2^). We measured species identity and diameter at breast height (dbh = 1.4 m) for all woody plants greater than two cm dbh. We then sub-sampled plots for smaller woody plants by randomly choosing a quadrant (177 m^2^) delimited by the north to south and east to west axes, and then counting all woody stems taller than 50 cm and <2 cm dbh within the quarter circular plot. Finally, we estimated woody seedling density in eight 1 m^2^ plots located on alternating sides of the cardinal axes of the plot. Seedlings were restricted to individuals of a tree species that were ≤50 cm in height.

To capture within site differences, we sampled two plots at most sampling locations, where plots were separated by at least 40 m. We collected four quantitative plots in a 2008 pilot study and an additional 24 in 2009, for a total of 28 quantitative plots (1.98 ha, total). In addition, we sampled 11 rapid survey plots, where trees and saplings were counted within size groupings and seedlings were not sampled. These rapid assessment plots were used to record composition around additional tree-ring sampled trees and are not included in our quantitative assessment of forest composition.

We focus our analysis on trees, defined here as woody plants that grow with a dominant trunk, have a distinct canopy (ie, not with continuous foliage from the ground to the top of the plant) and attain a size of 20 cm dbh in at least one of our sampled plots. Forest tree structure and composition were characterized by calculating the importance value (IV300) for each tree species encountered, where IV300 is the sum of the relative density (0–100), relative frequency (0–100), and relative basal area (0–100) [28]. Finally, we focused our sampling on stands that appeared undisturbed and contained conifers. Most hardwood dominated stands exhibited evidence of coppicing. Visual assessments confirmed that conifers were dominant across much of the lower valleys, interspersed with small pockets of hardwood dominated stands.

### Tree Radial Growth

Age structure was measured for trees only using tree-ring cores and dbh measurements. In total, 140 cores were collected from multiple taxa, with most cores taken from conifers in the genera *Pinus*, *Abies*, *Picea*, *Larix* and *Juniperus*. Cores were taken from mature trees with a maximum amount of their crown unshaded by neighbors. However, since most of JNNR is intact forest, most trees were shaded at some point in their canopy. One core per tree was sampled, on the mid-slope side of the tree at breast height.

The number of taxa used for tree-ring analyses was reduced to the four species (*Abies fargesii*, *Larix potaninii*, *Picea purpurea* and *Pinus tabuliformis*) with sample size (n) >10 (Σ(n) = 82 cores). Of these, *L. potaninii* and *P. tabuliformis* are considered early successional species, while *A. fargesii* and *P. purpurea* are considered late-successional species.

Tree cores were processed in the laboratory according to standard techniques [29]. Rings were crossdated visually using the list method [30] and with COFECHA [31], [32]. Radial ring widths were measured to the nearest 0.001 mm using a Velmex stage measuring system and accompanying MeasureJ2X software (Velmex, Inc., Bloomfield, NY, USA). Crossdating accuracy was double-checked after measurement using COFECHA; problematic cores were flagged and re-measured as necessary [31].

Standardization and chronology building were carried out using the dplR package for dendrochronological analyses [33] in R version 2.14.1 [34]. We used the interactive detrending feature in dplR to inspect curve fit of every core. Since the likelihood of endogenous disturbances (e.g. gap formation/closure) is high in our study area, many cores exhibited low frequency trends typical of these types of disturbances [35]. To remove these trends and maximize the climate signal, we applied a flexible smoothing spline of 30 years, which removes 50% of the variance at 30 years [36]. We also used autoregressive modeling [37] on each detrended chronology to minimize temporal autocorrelation among ring widths. To reduce the effect of outliers and further enhance the climate signal, we averaged the resulting standard chronologies together using a biweight robust mean [36]. One chronology per species was developed for the entire study area.

**Figure 2:**
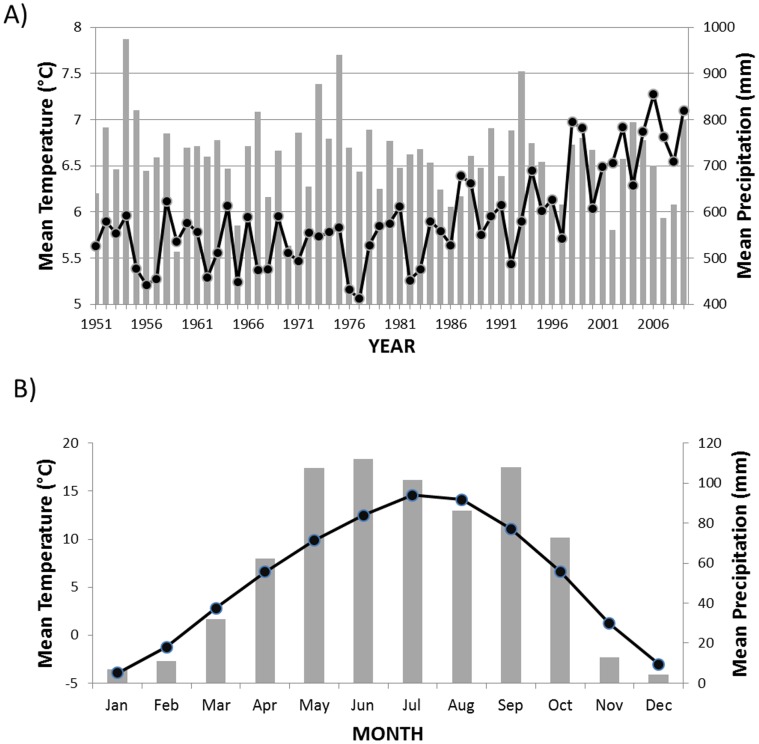
A regional climate summary for Jiuzhaigou National Nature Reserve. Annual (A) and monthly (B) climate summaries from the Songpan meteorological station (32.65N 103.56E, 2852 m elevation). This station is the longest-running station near Jiuzhaigou National Nature Reserve (JNNR). Mean temperature (dots; left axis) and precipitation (grey bars; right axis), 1951–2009. Data are from the Chinese Meteorological Association [37].

Climate data were obtained from the China National Meteorological Centre [38]. We downloaded monthly climatic data for mean temperature and precipitation from the Songpan climate station (location: 32.65N 103.56E, 2852 m elevation). This station is the closest long-term weather station to JNNR, and provided us with climate data for the region from 1951 to 2009 (Fig. 2).

Correlations between radial growth and climate were carried out using DENDROCLIM 2002 [39], using the residual chronologies produced by autoregressive modeling after detrending (see above). DENDROCLIM uses bootstrapped error estimates of correlation to test for significance instead of traditional linear approaches [39], [40]. A 21-month window, from January of the previous year through September of the current year, was analyzed for every climate-chronology pair of data trends for each species. Monthly correlation functions [35] were produced by DENDROCLIM 2002 for each pair over the 21-month period.

## Results

### Forest Structure: Conifer Versus Hardwood Dominance

**Figure 3:**
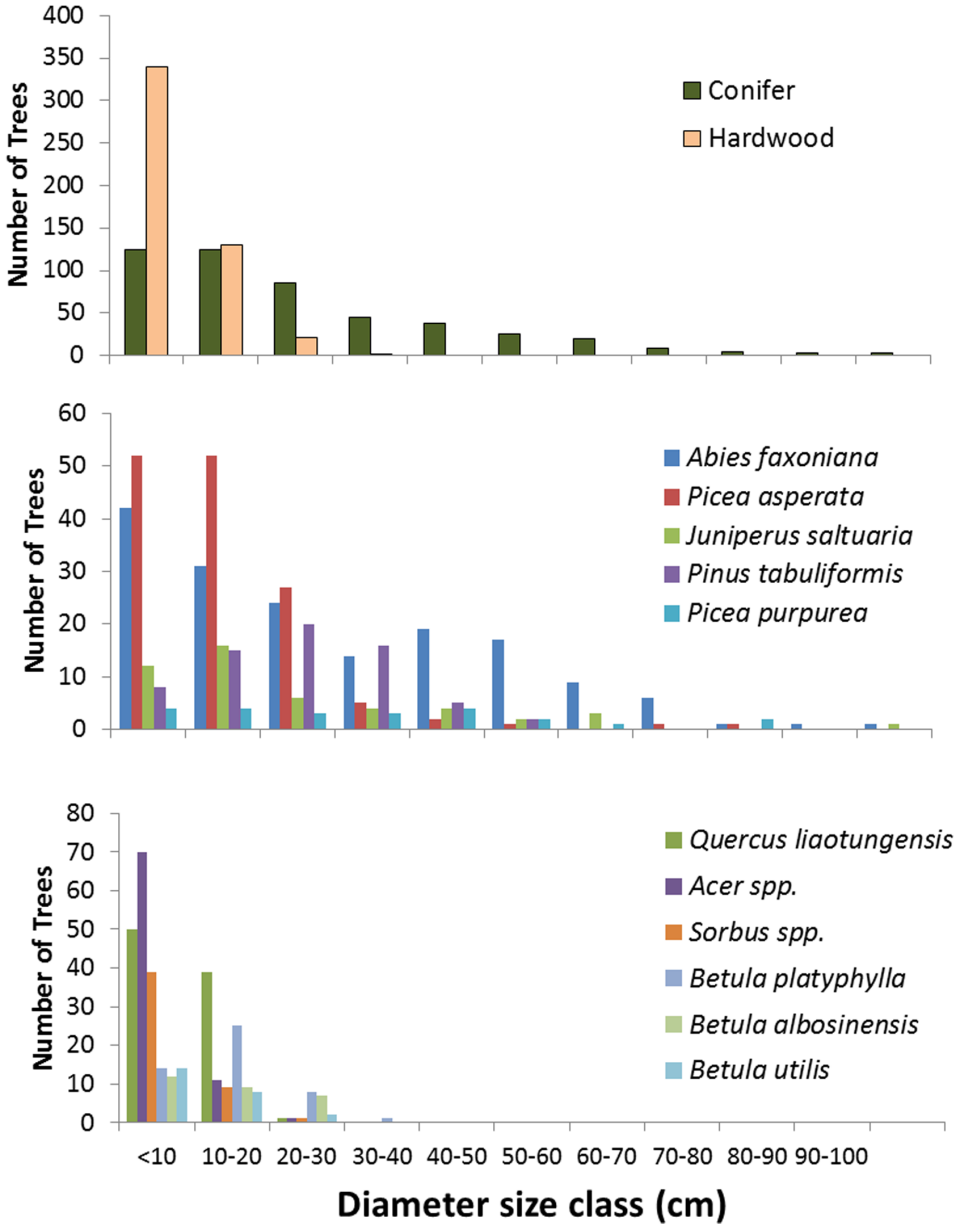
Forest composition for Jiuzhaigou National Nature Reserve. Tree diameter at breast height (dbh) class distributions in 39 forest plots (approximately 2.75 ha) distributed across the Jiuzhaigou National Nature Reserve. (A) Canopy trees divided into conifer and hardwood categories, showing a strong dominance by conifers among large diameter trees. (B) Diameter class distributions of the five most abundant conifer tree species. (C) Diameter class distributions among the dominant hardwood taxa, mostly defined to the generic level. Diameter class categories by 10 cm intervals for all trees greater than 2 cm dbh.

Vegetation plots were strongly dominated by conifers in the overstory and hardwoods in the sub-canopy, a pattern that is reflected in the distribution of tree type across diameter (dbh) classes (Fig. 3a). Hardwoods, often species that grow as shrubs or small sub-canopy trees, were generally dominant (i.e., >50% of individuals) in the understory and lower diameter classes. Nevertheless, conifers were present in all diameter classes and most abundant in larger classes, suggesting a steady level of recruitment. Among the 36 woody species or morpho-species encountered, six conifers from four genera (*A. fargesii, P. asperata, Juniperus saltuaria and P. tabuliformis, Picea. wilsonii, P. purpurea)* comprised 89% of total forest basal area, and 47% of the total number of trees counted (Table 1). All but three of our 39 plots were dominated in basal area by conifers, with conifers comprising an average of 84% of plot basal area.

**Table 1:** All woody taxa exceeding 2 cm dbh sampled in 28 circular 15 m radius forest plots of Jiuzhaigou National Nature Reserve (Sichuan Province, China).

Species	DensityNumber/ha	Basal Area m2/ha	Frequency # plots/28	IV 300	SaplingsNumber/ha	Seedlings Number/ha
a. Conifers						
*Abies fargesii*	83.37	9.17	0.75	70.97	36.38	12679
*Picea asperata*	71.24	2.18	0.32	29.88	4.04	5089
*Junpierus saltuaria*	24.25	2.03	0.32	19.67	10.11	0
*Pinus tabuliformis*	33.35	2.02	0.18	18.91	2.02	4196
*Picea wilsonii*	7.58	3.04	0.11	16.91	*	*
*Picea purpurea*	11.62	1.57	0.21	13.14	*	*
*Larix mastersiana*	5.56	0.38	0.14	5.36	0.00	179
*Pinus armandii*	3.54	0.04	0.07	2.18	*	*
*Abies ernestti*	2.53	0.15	0.04	1.82	*	*
b. Large hardwoods						
*Quercus liaotungensis*	45.47	0.40	0.21	14.86	24.25	625
*Quercus* spp. *(6)*	4.04	0.02	0.04	1.55	14.15	179
*Sorbus* spp. (12)	24.76	0.15	0.50	14.62	4.04	804
*Acer* spp. (13)	41.43	0.15	0.29	14.20	99.03	5536
*Betula platyphylla*	24.25	0.48	0.29	12.15	8.08	*
*Betula albosinensis*	14.15	0.25	0.25	8.43	22.23	714
*Betula utilis*	12.13	0.13	0.14	5.58	8.08	*
*Betula* sp. (6)	0.51	0.00	0.04	0.74	*	1071
*Fraxinus* sp. (3)	7.07	0.03	0.18	4.75	0.00	179
*Tilia* sp. (4)	10.61	0.10	0.11	4.51	0.00	179
*Prunus* sp.(4)	7.07	0.05	0.14	4.19	16.17	268
*Salix* sp. (32)	4.55	0.05	0.11	3.07	0.00	89
*Lonicera chrysantha*	2.53	0.01	0.04	1.18	0.00	0
*Lonicera* sp. (29)	1.52	0.01	0.04	0.98	0.00	0
*Berchemia floribunda*	2.02	0.02	0.04	1.14	2.02	0
Unknown hardwood	12.63	0.06	0.29	7.93	80.84	446
c. Small hardwoods						
*Rhododendron* sp. (34)	12.13	0.02	0.14	5.10	14.15	0
*Dipelta yunnanensis*	5.05	0.01	0.14	3.62	0.00	0
*Carpinus* sp. (2)	5.56	0.02	0.11	3.13	14.15	0
*Cretaegus kansuensis*	2.02	0.00	0.07	1.70	12.13	89
*Helwingia japonica*	2.02	0.00	0.07	1.69	129.34	536
*Viburnum* spp. (15)	2.02	0.00	0.07	1.69	10.11	89
*Viburnum cylindricum*	1.52	0.00	0.04	0.96	*	89
*Clematoclethra* sp. (3)	1.01	0.02	0.04	0.95	0.00	89
*Litsea chunii*	1.01	0.00	0.04	0.85	0.00	0
*Cornus* sp. (2)	1.01	0.00	0.04	0.85	74.78	1607
*Ostryopsis davidiana*	0.51	0.00	0.04	0.74	0.00	0

* seedlings and saplings not distinguished within genera and for the purpose of this table lumped into the most abundant taxa identified within the genus.

Plots sampled cover a total of 1.98 sampled hectares. Total stem count, total basal area sampled, and the frequency of species encountered in plots are presented along with Importance Value (IV300: sum of relative stem density, relative basal area and relative frequency). Species are ordered by IV 300 within classes of : (a) conifers; (b) hardwoods that somewhere exceed 10 cm dbh; and (c) hardwoods that never exceed 10 cm dbh in our sample (including species that primarily grow as shrubs and vines). Seedling and sapling counts are from sub-samples within forest plots. Saplings were counted in one quadrant of the plot (a randomly chosen 90° quarter circle, 177 m^2^). Seedlings were counted in eight 0.5 m^2^ subplots within each plot. Parenthetical numbers following generic designations indicated the number of recognized species found within JNNR [18].

In aggregate, sampled plots exhibited the diameter class structure that we would expect in a stably regenerating forest, with fewer individuals in each larger diameter class (Fig. 3a). Within species, size distribution patterns varied: the dominant conifers, *A. fargesii* and *P. asperata* both exhibited the inverse J-shaped curve indicative of regenerating forests; while *J*. *saltuaria*, *P. tabuliformis* and *P. purpurea* all showed a dearth of individuals in the smallest diameter classes (Fig. 3b). Similarly, the sub-canopy hardwoods *Quercus liaotungensis* and the maples (*Acer* spp.) showed declining densities of larger individuals with increasing diameter (Fig. 3c).

The sapling and seedling data generally reflected the forest overstory patterns, with the sapling strata dominated by small-statured hardwood trees and shrubs and smaller numbers of conifer saplings (Table 2). Conifer saplings were found most frequently in stands with a high abundance of small conifer trees (*A. fargesii, Picea spp.*; Table 1). This pattern also held in the seedling stages with *A. fargesii* being the most prevalent species observed, followed by maple (*Acer* spp.), spruce (*Picea* spp.), and pine (*P. tabuliformis*) seedlings (Table 1).

**Table 2:** Tree-ring descriptive statistics for four conifer species from Jiuzhaigou National Nature Reserve used in dendroclimatological analyses.

Taxon	Sample Size (# of trees,1 core/tree)	Mean Sensitivity*	Inter-Series Correlation†	Avg. Sample Length (Years)
*Abies fargesii*	22	0.165	0.304	129.2
*Larix potaninii*	11	0.213	0.405	160.2
*Picea purpurea*	23	0.154	0.369	217.5
*Pinus tabuliformis*	25	0.208	0.382	120.2

* Mean sensitivity is the average of the relative difference in widths of adjacent rings for all cores [34].

† Inter-series correlation is the average of all correlation for all series (cores) within a chronology or species.

*Pinus tabuliformis* and *Larix potannii* are considered pioneer, shade intolerant species. *Picea purpurea* and *Abies fargesii* are more shade tolerant and can be found in old-growth forest. Statistics were produced by COFECHA during cross-dating.

**Figure 4:**
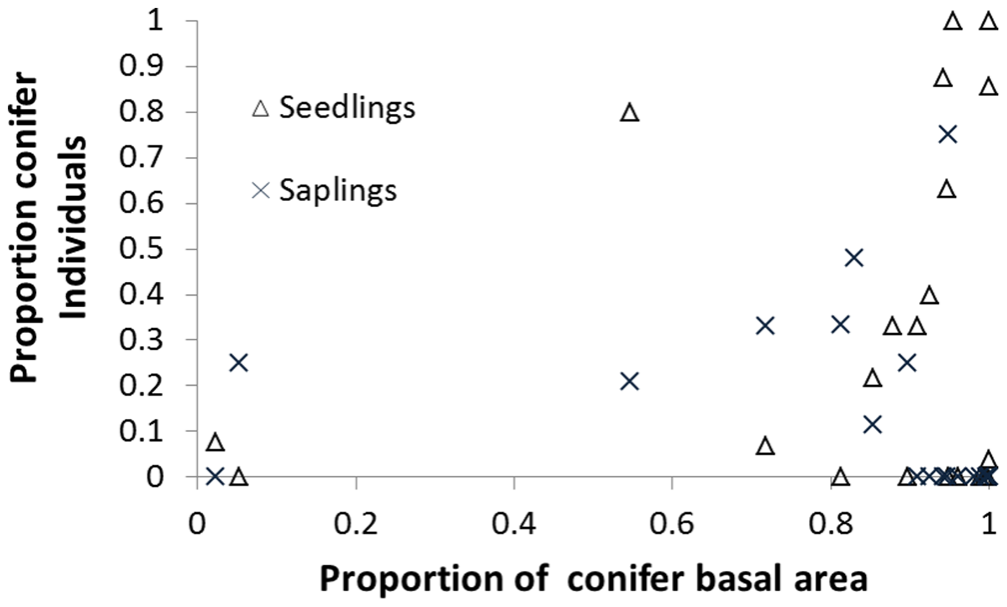
Conifer recruitment as a function canopy dominance. Twenty-eight forest plots within Jiuzhaigou National Nature Reserve plotting the proportion of conifer seedlings and saplings as a function of the proportion of total basal area accounted for by conifers. While the canopy of most plots consisted primarily of conifers, seedlings and/or saplings in these plots were not necessarily predominantly conifers. Plots with low abundance of conifers in the overstory generally had low abundance of conifer seedlings and saplings.

The proportion of conifer trees in a plot was weakly related to the proportion of conifer seedlings and saplings (Fig. 4). Although plots that had mostly hardwood trees did not have many conifer seedlings or saplings, plots that had mostly conifer trees had a mix of species represented by seedlings. A strong majority (70%) of plots that had a majority of conifer trees had no conifer saplings, while about half of those plots had some conifer seedlings. Total plot basal area and the proportion of conifer trees each had non-significant negative correlations with total seedlings, total saplings, and the fraction of seedlings or saplings that were conifers (all p>0.05).

### Forest Structure across a Climate Gradient

**Figure 5:**
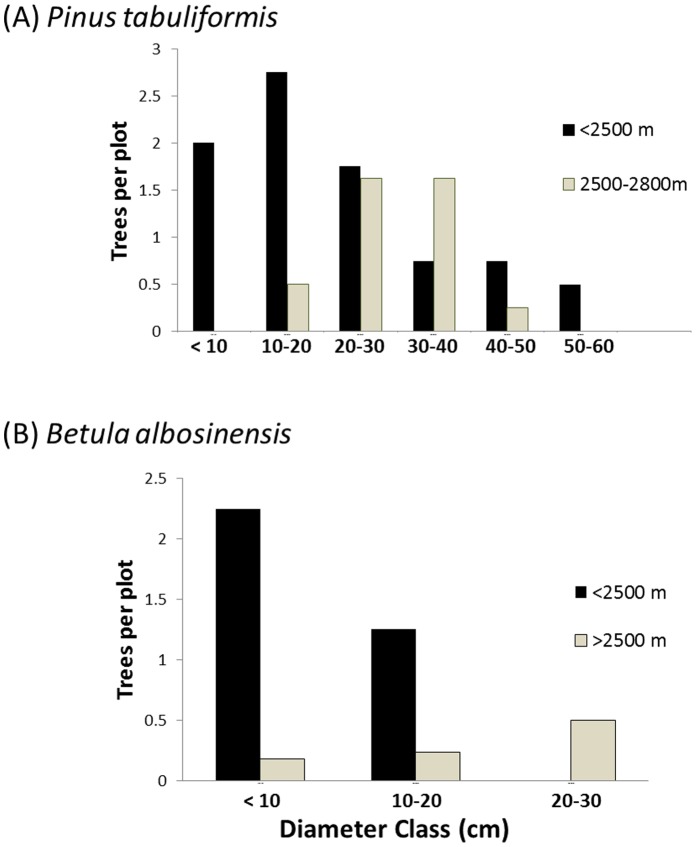
Diameter class distributions of dominant trees in Jiuzhaigou National Nature Reserve by elevation. Plots of (A) overstory trees of *Pinus tabuliformis* and (B) sub-canopy trees of *Betula albosinensis* in, segregated into elevation class. *P. tabuliformis* was found in 5 of 28 sampled plots, including 3 of the 4 plots sampled in the lowest elevation class (2275– 2500 m), and 2 of the 8 plots in the next lowest elevation category (2500–2800 m). *B. albosinensis* was found in 7 of 28 plots, including 1 of the 4 plots in the lowest elevation class and 6 of 17 plots between 2500 and 3100 m.

Assessing plot structure across an elevation gradient that reflects changing climatic conditions (i.e., temperature, precipitation) revealed no patterns of increasing hardwood or conifer presence at any strata (overstory, sapling or seedling). Average tree diameter, however, increased significantly with elevation (n = 28, r = 0.62; p<0.001). Individual species exhibited differences in abundance across elevation, but generally did not differ markedly in diameter class structure across elevation (data not shown). *P. tabuliformis* was the exception, showing a trend of having mostly small diameter trees in lower elevation plots (<2500 m), and mostly large diameter trees in higher elevation plots (2500–2800 m; Fig. 5a). Birches (*Betula* spp.) showed a mixed pattern with respect to diameter distribution across elevation. Specifically, all seven *B. albosinensis* individuals greater than 30 cm dbh were found in moderately high elevation plots (2800 and 3100 m), whereas 75% of individuals <10 cm dbh were found below 2500 m elevation (Fig. 5b). In contrast, all 14 small (<10 cm dbh) *B. platyphylla* trees were sampled at moderately high elevation (2800 and 3100 m), while 78% of the largest trees (>20 cm) were found below 2500 m elevation. *B. utilis* was found exclusively above 3100 m and conformed to the expectation of decreasing numbers of trees with increasing diameter class (data not shown). Finally, *B. utilis* was only found above 3100 m elevation.

### Radial Growth and Correlation with Climate

**Figure 6:**
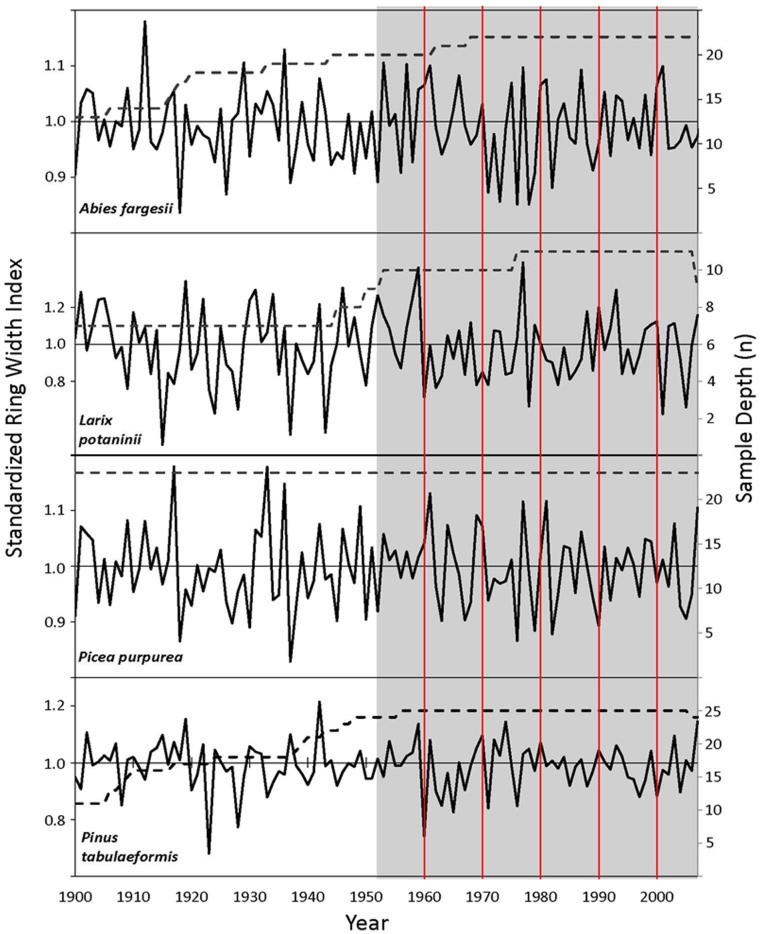
Standard tree-ring chronologies for four conifers of Jiuzhaigou National Nature Reserve. Chronologies span 1900–2007 for the four species (*Abies fargesii*, *Larix potaninii*, *Picea purpurea* and *Pinus tabuliformis*) used in dendroecological analysis. The ring width index is represented by the solid line (left axis) and sample depth by the dashed line (right axis). The period (1952–2007) analyzed for growth-climate correlation function is highlighted in grey. To facilitate alignment across species, four vertical lines are placed at 1960, 1970, 1980, 1990 and 2000. See Methods for more detail on how chronologies were built.

Interseries correlation for radial growth as measured in tree-rings ranged from 0.3 to 0.4 for the four species analyzed (*A. fargesii*, *L. potaninii*, *P. purpurea,* and *P. tabuliformis*). Mean sensitivity was highest for *L. potaninii* and *P. tabuliformis* (Table 2). Detrending produced residual chronologies with a stable mean and variance over the 1952 to 2007 period analyzed for climate-growth analyses (Fig. 6), with a few years showing responses common across all four species (e.g. 1961 and 1977).

**Figure 7:**
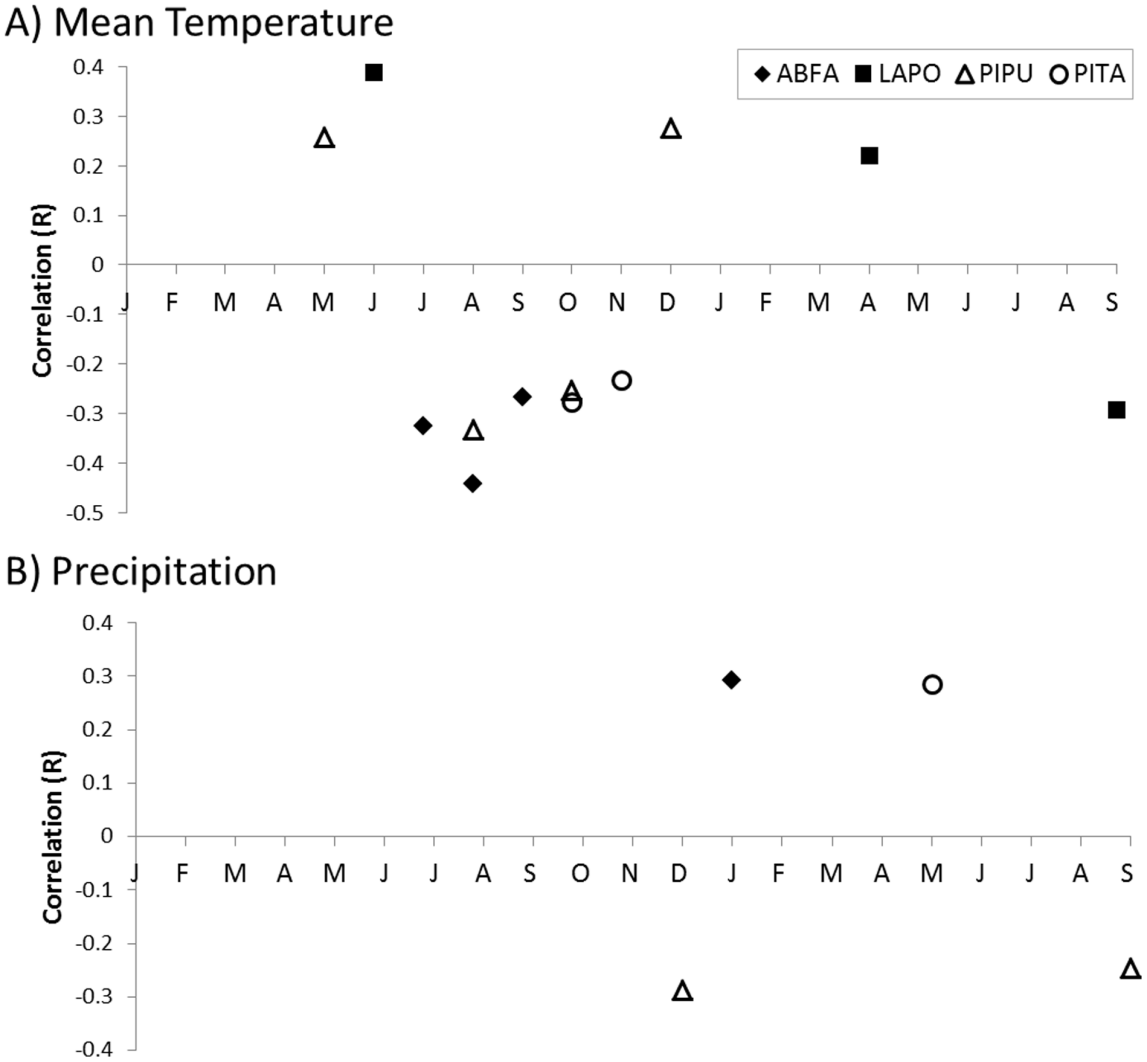
Correlation function analysis of tree growth to climate for trees of Jiuzhaigou National Nature Reserve. Results of growth and (A) mean temperature, and (B) precipitation for the 21-month period of time starting in January of the previous year through September of the current year’s growth from 1952 to 2007. See Fig. 2 for graphical summary of climate data used in this analysis [37]. Only significant values at *p* = 0.05 are shown. Species codes as shown in the legend are ABFA (*Abies fargesii*), LAPO (*Larix potaninii*), PIPU (*Picea purpurea*) and PITA (*Pinus tabuliformis*).

Overall, the four species analyzed were more responsive to fluctuations in temperature than precipitation (Fig. 7). Radial growth of *A. fargesii* was significantly negatively correlated with mean temperature July through September of the previous year, and significantly positively correlated with precipitation in January of the current year. *L. potaninii* was significantly positively correlated with temperature in June of the previous year and April of the current year, as well as September of the current year; there were no significant correlations with precipitation. *P. purpurea* was significantly negatively correlated with temperature in August and September of the previous year, but significantly positively correlated with temperature in May and December of the previous year. *P. purpurea* was also significantly negatively correlated with precipitation in December of the previous year and September of the current year. *P. tabuliformis* was significantly negatively correlated with temperature, only in October and November of the previous year, and with precipitation in May of the current year.

## Discussion

Our rapid assessment depicts current stand composition. Tree-ring data add historical depth through measures of growth rates. Our tree core sampling, however, was not in sufficient detail to allow us to infer past stand structure. We can say, however, that the cumulative numbers of conifers distributed across diameter classes suggest (though they do not conclusively show) the kind of steady recruitment that can maintain current stand structure into the future. Thus, even though a majority of conifer-dominated stands was characterized by a hardwood sub-canopy and had numerically more hardwood saplings and seedlings than conifer ones, we cannot conclude that this pattern reflects a transition from conifer- to hardwood-dominated tree canopies; nor can we rule out that the current sub-canopy dominance by hardwoods under an overstory dominated by conifers is a temporally stable structure for these forests. Lacking temporal data, our plots provide only weak capacity to distinguish among these options.

Still, we can assess what we would expect to see if plot structure implied a forest in transition as a consequence of changing climates. If warming climates were driving upslope distribution shifts, we would expect to find a trend of increasing prevalence of smaller, younger individuals for a given species with increasing elevations. With the exception of *B. platyphylla*, this is not the case. In fact, both *B. albosinensis* and *P. tabuliformis* exhibit diameter structure by elevation patterns that suggest downslope shifts (i.e., more small-diameter trees at lower elevation; Fig. 5). Given that we observed an increase in tree diameter with elevation, and most (34 of 38) of the largest trees (>60 cm dbh) were observed above 2800 m in elevation, this implies that forests at lower elevations may have been more frequently disturbed. Most of the human influence on forests in terms of tree harvesting has occurred below 2800 m, according to local forest department records (personal communication with park rangers). As such, patterns of downslope shifts may simply reflect recovery from previous harvest pressure. Supporting the contention that shifts in *P. tabuliformis* are not climate driven is the observation that this species exhibited no particular growth response in tree-rings through time and little correlation with climatic signals.

Under a hypothesis of climate change response, we might expect to find disparity between the seedling, sapling and small understory tree composition, relative to the overstory composition, or by species relative to elevation. That is, because the climatic conditions for establishment today are different than they were when today’s mature trees established themselves, one might expect that a different suite of species would be favored. But such is not the case. We find few significant relationships between seedling density, sapling density, seedling composition or sapling composition with the fraction of conifer overstory trees, or relative to elevation. Those that we did find (e.g., fewer hardwood seedlings with increasing elevation) do not suggest a warming response. Thus, we do not find structural evidence for change in canopy composition or evidence of poor or declining conifer recruitment that may be indicative of stress and competitive displacement by faster growing hardwoods. Similarly, others have also found a stably coexisting mixed conifer and hardwood forest composition in montane environments of the same region [41]. Our conclusions should be considered preliminary because they are based on a static view of forest pattern. A detailed demographic study would be required to rule out the hypothesis of a climate change response among trees of this region.

Radial growth of the four conifer species we analyzed (*A. fargesii*, *L. potaninii, P. purpurea,* and *P. tabuliformis*) appears to be more tied to temperature than precipitation, especially temperature during summer and fall of the previous year (Fig. 7). Most precipitation in the region occurs in synch with the growing season (Fig. 2) and thus may not usually be a limiting factor in growth. Growth of *A. fargesii*, has previously been shown to be more tied to temperature than precipitation [3], [42]. Fan et al. [43] concluded that radial growth of four species of *Abies* and *Picea* from a nearby mountain system in the Tibetan Plateau with a similar, humid growing-season climate, was most consistently limited by temperature during November of the previous year. Studies have shown *P. tabuliformis* to be generally non-responsive to temperature variation, but positively correlated with precipitation during the growing season [43]–[45]. In JNNR, *P. tabuliformis* is found at lower elevations and on drier sites, and thus, might be expected to be more sensitive to precipitation than other conifers in our study. However, our data suggest it is no more sensitive to precipitation than other conifers at JNNR.

Our results suggest that temperature during the growing season, extending into fall, (July – Nov.; Fig. 7) may be the most limiting aspect of climate on growth for conifer species in JNNR. In a study on *A. fargesii* (AKA *A. faxoniana*) from the nearby Wanglang Nature Reserve, Zhao et al. [46] similarly concluded that growth was negatively correlated with temperature during the previous growing season (they also found a positive correlation with precipitation in January, as we did). Previous studies have emphasized the importance of temperature during winter, fall and spring in limiting radial growth, but also observed non-significant negative correlations or response functions between growth and summer temperatures [42], [43]. Conifers in JNNR appear to prefer cool summer temperatures for good radial growth. This result may represent a moisture availability, or drought stress signal. Despite our low correlation between growth and precipitation, drought stress likely has an impact on growth of trees in the region. Several studies from central and SE China have shown high correlations between Palmer Drought Severity Index (PDSI) and growth [5], [8], [43], [46]. Also, growth of *P. tabuliformis* has been shown to be sensitive to drought in northern China [6], [44], [45].

Temperature data from the closest long-term station to JNNR indicate that the climate has warmed steadily since the early 1980s (Fig. 2). If this trend continues, how conifers of JNNR will respond, at least in terms of radial growth, probably depends on the season of change and precipitation. Assuming steady precipitation, our data suggest that conifer species would respond positively to increased warmth in winter and spring, but negatively to increased warmth in the summer and fall. Response is not likely to be consistent across all of JNNR for a given species. Several studies have demonstrated variable growth response to climate, depending on topographic position [3], [5], [43], [46].

### Conclusions and Future Directions

Our results on forest stand structure, in association with simple observations in the field (e.g., coppiced trees, stumps, even-aged planted stands), depict a forest recovering from 20^th^ century timber and fuelwood extraction. These earlier land uses make definitive prediction of stability or transition difficult. Nevertheless, stand structure is generally consistent with what could be interpreted as replacement levels of conifer regeneration within conifer dominated regions. Our results on growth-climate relationships of four JNNR conifers is roughly in line with many other recently published studies from nearby regions, but suggests that conifers of JNNR may be particularly susceptible to warming during the growing season. Future studies should examine change during this crucial period more thoroughly, including data that incorporate both precipitation and temperature (e.g. PDSI). An analysis of growth-climate relationships across gradients of topography would also be prudent. These types of studies are becoming more common in dendrochronology [47], and would aid JNNR staff and their understanding of how and where to focus conservation efforts of the park’s tree species. Finally, to date, very little has been reported about radial growth of *P. purpurea* (though see [48]) despite its relatively widespread distribution in subalpine regions of central China. This species is long-lived, common in JNNR and demonstrated a strong sensitivity to climate variability in our data.
